# A meta-analysis of serum osteocalcin level in postmenopausal osteoporotic women compared to controls

**DOI:** 10.1186/s12891-019-2863-y

**Published:** 2019-11-13

**Authors:** Zhongyu Liu, Ruiqiang Chen, Yutong Jiang, Yang Yang, Lei He, Chunxiao Luo, Jianwen Dong, Limin Rong

**Affiliations:** 1 Department of Spine Surgery, Institute of Drug Clinical Trial for Orthopedic Diseases, The Third Affiliated Hospital of Sun Yat-Sen University, Guangzhou, 510630 China; 2 Department of Rheumatology, The Third Affiliated Hospital of Sun Yat-Sen University, Guangzhou, 510630 China

**Keywords:** Osteocalcin, Bone turnover marker, Postmenopausal osteoporosis, Meta-analysis

## Abstract

**Background:**

Circulatory osteocalcin (OC) has been widely used as a biomarker to indicate bone turnover status in postmenopausal osteoporosis (PMO). However, the change of serum OC (sOC) level in PMO cases compared to postmenopausal controls remains controversial.

**Methods:**

We searched the online database of PubMed and Cochrane Library. A meta-analysis of case-control studies was performed to compare the pooled sOC level between PMO patients and postmenopausal controls. Subgroup analysis according to potential confounding factors (different OC molecules and regions of the study population) was also performed.

**Results:**

Ten case-control studies with 1577 postmenopausal women were included in this meta analysis. We found no significant difference in the pooled sOC level [mean difference (MD) = 1.84, 95% confidence interval (CI): (− 1.49, 5.16), *p* = 0.28] between PMO patients and controls. Subgroup analysis also revealed no significant difference in intact OC [MD = 1.76, 95%CI: (− 1.71, 5.23), *p* = 0.32] or N-terminal mid-fragment of the OC molecule [MD = 0.67, 95%(− 5.83, 7.18), *p* = 0.84] between groups. For different regions, no significant difference in sOC was found in Asian population between cases and controls [MD = -0.06, 95%(− 6.02, 5.89), *p* = 0.98], while the pooled sOC level was significantly higher in European PMO cases than controls [MD = 3.15, 95%(0.90, 5.39), *p* = 0.006].

**Conclusions:**

Our analysis revealed no significant difference in sOC level between PMO cases and controls according to all the current eligible studies. OC molecules are quite heterogeneous in the circulation and can be influenced by glucose metabolism. Therefore, sOC is currently not a good indicator for the high bone turnover status in PMO. More trials with standardized methodologies for the evaluation of circulatory OC are awaited to update our current findings.

## Background

Osteoporosis (OP) is a systemic skeletal disorder characterized by low bone mass and deteriorated microarchitecture of bone tissue. The osteoporotic bone is fragile and susceptible to fracture [1]. Postmenopausal osteoporosis (PMO) results from estrogen deficiency after menopause and is a major type of primary osteoporosis [2]. PMO may lead to fragility fracture and is one of the most disabling consequences of postmenopausal women. Early identification and effective therapeutic monitoring to PMO is necessary to reduce the public health burden of this pervasive disease [3].

Osteocalcin (OC), also known as the bone Gla protein (BGP), is a 5.8 kDa, hydroxyapatite-binding protein that could be synthesized by osteoblasts, odontoblasts and hypertrophic chondrocytes [4] and is the most abundant non-collagenous protein found in bone matrix. On the one hand, OC molecules can be released directly into blood after osteoblastic synthesis during bone formation; on the other hand it can also enter the circulation from osteoclastic bone matrix degradation during bone resorption. Therefore, circulatory OC may come from both bone formation and bone resorption [5, 6] and serum osteocalcin (sOC) level may theoretically increase in PMO, which is characterized by high bone turnover status with both increased bone formation and bone resorption.

Although serum osteocalcin (sOC) has been widely used as a bone turnover marker to indicate the high bone turnover status in PMO [7–9], the change of sOC level in PMO cases compared to controls remains controversial. Some of the previous studies have found a higher sOC level in PMO patients than age-matched controls, which is consistent with the high bone turnover status in PMO cases [10–12]. Meanwhile, there are also studies reporting the same or even lower sOC level in PMO cases than controls. In 2012, Biver et al. [13] performed a meta-analysis to compare several bone turnover markers between osteoporotic cases and controls and found no significant difference in sOC, which is so far the only pooled evidence of sOC change in OP. However, they included all types of osteoporosis where bone metabolic patterns might be quite heterogeneous. More studies have emerged since then and the pooled evidence of sOC change in PMO cases compared to controls is needed.

To obtain the evidence, we performed a meta-analysis of the available case-control studies to compare the sOC level between PMO patients and age-matched postmenopausal controls.

## Methods

We followed the Preferred Reporting Item for Systemic Review and Meta-analysis (PRISMA) [14, 15] and performed this meta-analysis based on a protocol, according to the recommendations of the Cochrane Collaboration.

### Data sources and search strategies

We searched PubMed and Cochrane Library online for all the available case-control studies published up to (12 October 2018), without restriction to regions and languages. Detailed strategy was: (“biological markers”[Title/Abstract] OR “turnover markers”[Title/Abstract] OR “osteocalcin”[Title/Abstract] OR “BGP”[Title/Abstract]) AND (“postmenopausal osteoporosis”[Title/Abstract] OR “type I osteoporosis”[Title/Abstract]). Conventional searches were supplemented by manual searches of the reference lists of all the relevant studies, review articles and conference abstracts.

### Inclusion and exclusion criteria

Studies were included if they met the following inclusion criteria: 1.case-control study design; 2.patients in the case group were diagnosed of PMO, according to the diagnostic criteria recommended by WHO [16]; 3.participants in the control group were age and sex matched with cases; 4.sOC level was reported. Studies were excluded if they met the following exclusion criteria: 1.secondary osteoporosis, or under any circumstance that bone turnover or sOC level might be affected (e.g., hyperparathyroidism, diabetes mellitus, liver disease, renal insufficiency, anti-osteoporotic therapy, and long-term corticosteroid therapy); 2.studies including males or non-postmenopausal females.

### Study selection and data extraction

Two literature reviewers evaluated the eligibility of potential titles and abstracts independently. Included studies were reassessed as full text by inclusion and exclusion criteria. Disagreement was solved by discussion. Further adjudication of a third reviewer was performed if the disagreement remained. The following data were then extracted from each eligible study: the first author’s name, year of publication, demographic information (age, region, and number of people in case and control groups), sampling condition, manufacturer of the OC assay kit, target fragment of the sOC molecule and sOC level.

### Quality assessment of the included studies

Assessment to the risk of bias of case-control studies was performed using the Newcastle-Ottawa Scale (NOS) [17], as recommended by Cochrane Collaboration. A score of 0–9 was allocated to each study. Studies achieving six or more points were considered of high quality, which is essential for a high-level pooled evidence. Again, two reviewers independently evaluated the included studies and disagreement was solved by discussion. Further adjudication of a third reviewer was performed if the disagreement remained.

### Statistical analysis

The pooled sOC level was compared between cases and controls. Statistical analysis was performed using Review Manager (RevMan 5.3) software. First, heterogeneity of the included studies was tested. Heterogeneous data between studies was indicated by *p* ≤ 0.10 (or I^2^ ≥ 50%), and homogeneous data was indicated by *p* > 0.10 (or I^2^ < 50%). A Fixed Effect model was used for homogeneous data, whereas a Random Effects model was used for heterogeneous data. Continuous variables of sOC level were reported with 95% confidence interval (95% CI) of the mean difference (MD) and *p* value. The test of overall effect with a *p* < 0.05 was considered to be statistically significant. Collected data were carefully inputted, and then rechecked by two reviewers respectively.

Subgroup analysis according to different fragments of the sOC molecules and different regions of the study population was also performed, with the same method mentioned above. Heterogeneous data between subgroups was indicated by *p* ≤ 0.10 (or I^2^ ≥ 50%) and homogeneous data between subgroups was indicated by *p* > 0.10 (or I^2^ < 50%).

## Results

### Study selection

**Fig. 1:**
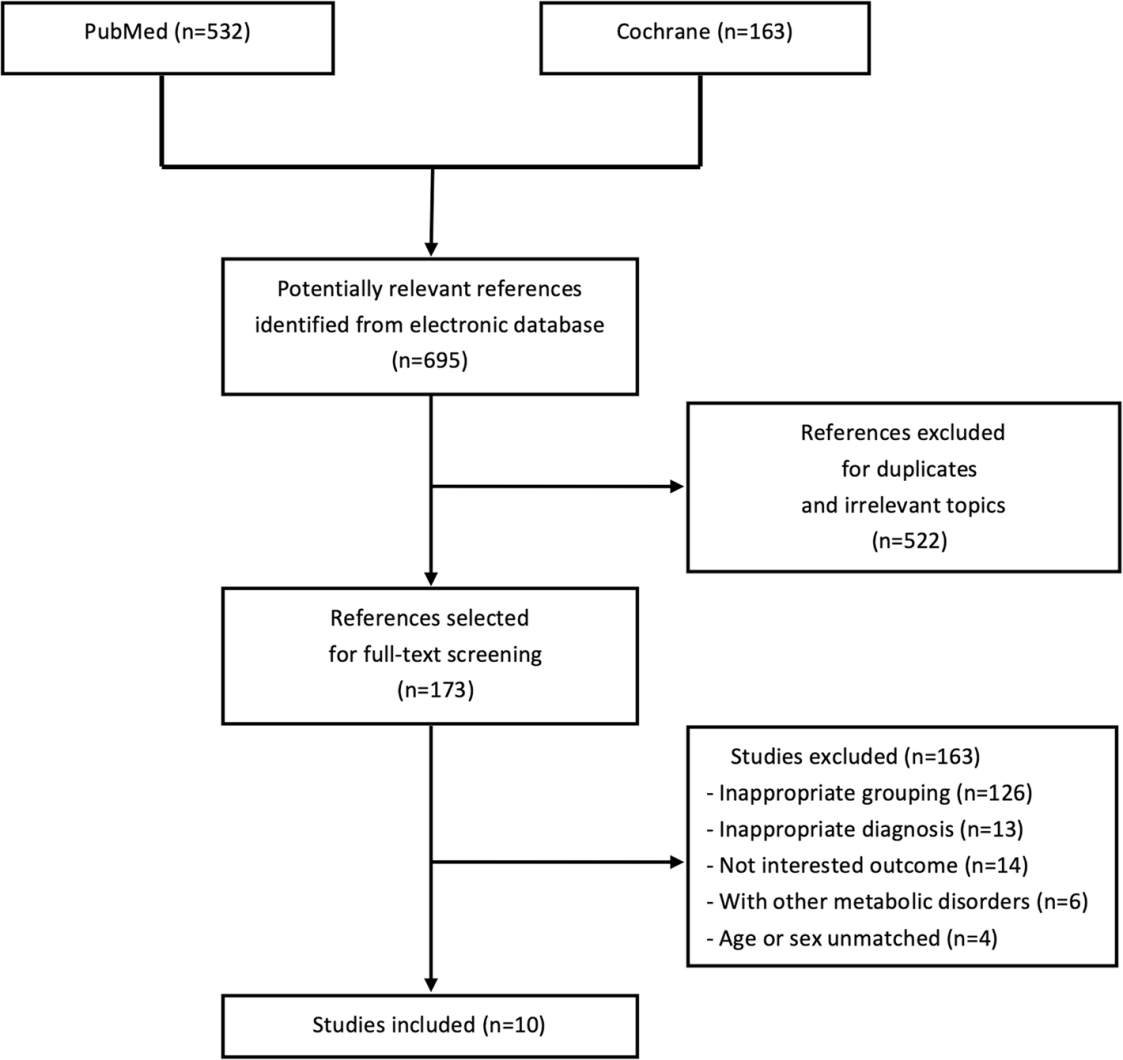
Flow chart of study selection. PMO, postmenopausal osteoporosis

Six hundred and ninety five references were identified, of which 10 studies [18–27] fulfilled all the inclusion criteria and were finally enrolled in this analysis, including 849 PMO patients and 728 postmenopausal controls (Fig. 1).

### Characteristics of included studies

Characteristics of the included studies are summarized in Table 1. The enrolled studies were all published in English, from China (2 studies), Saudi Arabia (1 study), Korea (1 study), UK (1 study), Turkey (2 studies), France (1 study) and Spain (2 studies), including Asian and European populations. Eight studies reported the assay kits used for OC evaluation. The assay kits from Roche targeting the N-terminal mid-fragment of OC molecule (N-MID) were used in 4 studies, while the kits from DSL, Metra Biosystems and Cis-bio detecting intact OC molecules and were adopted by 1 study respectively. Information of the definite target segment of OC molecule was not provided in the instructions of the kit from Incstar. Nine out of the ten studies reported fasting blood sampling. Serum OC level was evaluated and compared between PMO and control groups in each individual study. There was no significant difference in the mean age between cases and controls in all the enrolled studies.

**Table 1:** Characteristics of the included studies

Study	Country	Region	Sample size		Age (years)^a^		Manufacturer of the OC assay kit	Target^b^	Sampling condition	NOS^c^
			PMO	Control	PMO	Control				
Zhang 2015	China	Asia	257	90	62.73 (3.94)	61.36 (3.75)	Roche	N-MID	NA	6
Al-Daghri 2014	Saudi Arabia	Asia	100	100	50.6 (8.2)	48.6 (7.3)	Roche	N-MID	fasting	9
Jabbar 2011	UK	Europe	185	185	62.06 (14.53)	62.56 (13.24)	Roche	N-MID	fasting	9
Verit 2006	Turkey	Europe	45	55	55.68 (5.58)	55.21 (6.21)	NA	NA	fasting	7
Pouilles 2006	France	Europe	60	120	52.2 (2.5)	52.2 (2.7)	Roche	N-MID	fasting	9
Luo 2006	China	Asia	45	44	56.1 (4.4)	55.6 (5.9)	DSL	intact	fasting	9
Duman 2004	Turkey	Europe	75	66	53.16 (1.31)	52.62 (1.69)	NA	NA	fasting	7
Dominguez 1998	Spain	Europe	26	17	59 (6)	56 (7)	Metra Biosystems	intact	fasting	7
Kim 1996	Korea	Asia	14	37	56.2 (1.7)	55.6 (1.3)	Incstar	NA	fasting	7
Diaz 1995	Spain	Europe	42	14	62 (11)	“paired”	Cis-bio	intact	fasting	7

*OC* osteocalcin, *N-MID* N-terminal midfragment of osteocalcin molecule, *intact* intact osteocalcin molecule, *PMO* postmenopausal osteoporosis, *NOS* Newcastle-Ottawa Scale, *NA* data not available. ^a^Age is demonstrated with Mean (Standard deviation); ^b^Target fragment of the OC molecule; ^c^Range: 1–9. Studies achieving six or more points are considered of high quality

### Quality assessment

Quality assessment of the included studies was summarized in Table 1. All the 10 studies scored ≥6 points and were considered of high quality.

### Outcomes

#### Overall difference in sOC level between PMO cases and controls

**Fig. 2:**
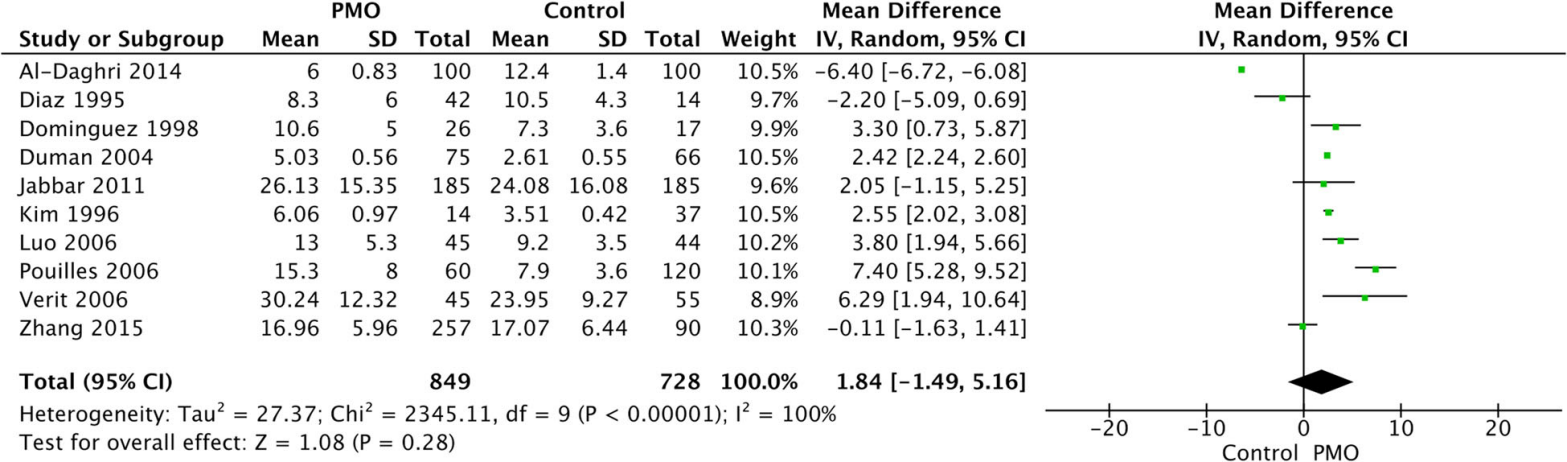
Forest plot of sOC level comparison between PMO patients and controls. sOC, serum osteocalcin; PMO, postmenopausal osteoporosis

Significant heterogeneity was indicated between studies (I^2^ = 100%). We therefore pooled the sOC level from all the included studies with Random Effects model. A trend of increased sOC level in PMO cases could be observed, while the difference in sOC level between cases and controls was not statistically significant [MD = 1.84, 95%CI: (− 1.49, 5.16), *p* = 0.28] (Fig. 2).

#### Subgroup analysis of the sOC level between PMO cases and controls

**Fig. 3:**
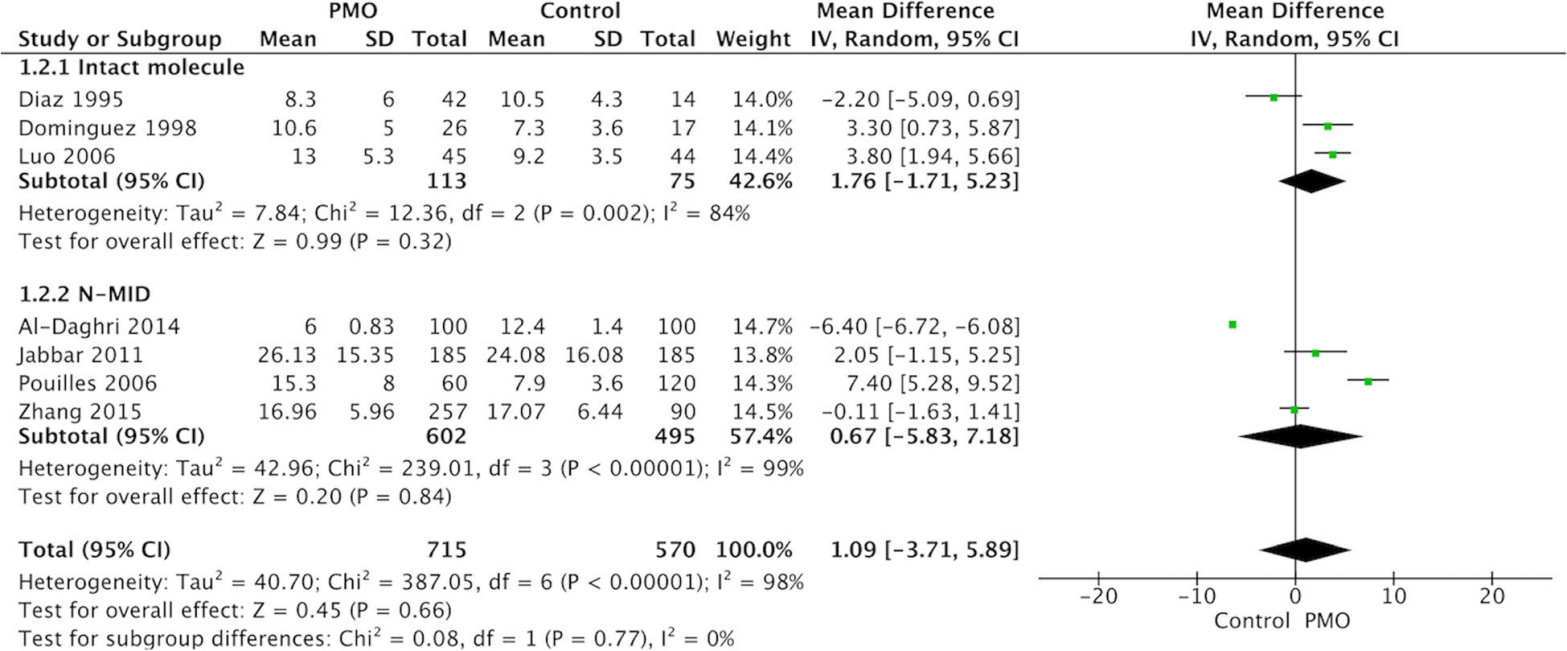
Subgroup analysis of different sOC molecules evaluated. sOC, serum osteocalcin; PMO, postmenopausal osteoporosis

**Fig. 4:**
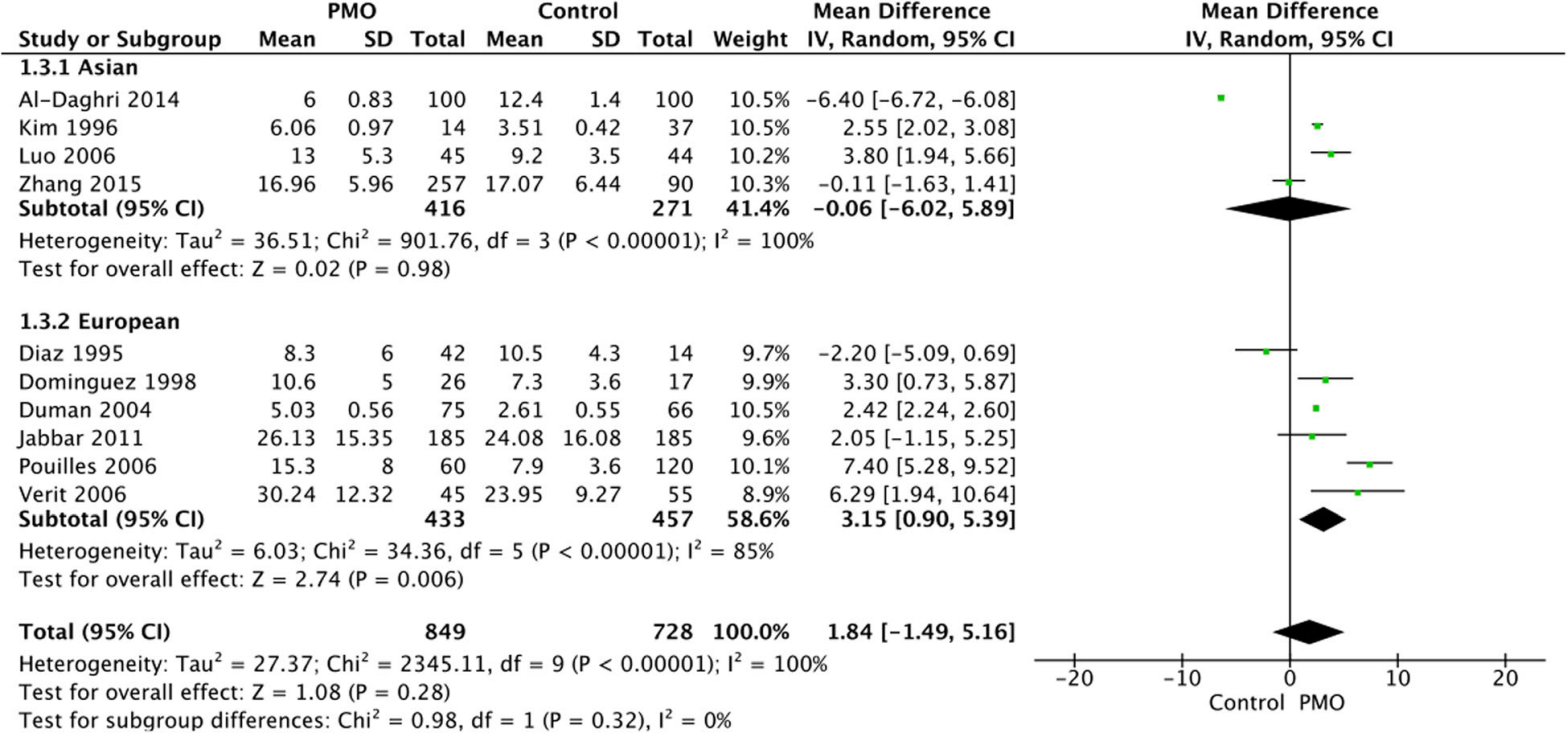
Subgroup analysis of different regions of the study population. PMO, postmenopausal osteoporosis

Subgroup analysis according to different target fragments of OC molecules revealed no significant difference in both intact sOC [MD = 1.76, 95%CI: (− 1.71, 5.23), *p* = 0.32] and N-MID level [MD = 0.67, 95%CI: (− 5.83, 7.18), *p* = 0.84] between cases and controls (Fig. 3). Meanwhile, subgroup analysis according to different regions demonstrated a significant increased sOC level in European [MD = 3.15, 95%CI: (0.90, 5.39), *p* = 0.006] but not in Asian PMO patients compared to controls [MD = -0.06, 95%CI: (− 6.02, 5.89), *p* = 0.98] (Fig. 4).

### Sensitivity analysis

We removed one study at a time and re-compared the pooled sOC level between cases and controls. Significant change occurred when removing Al-Daghri’s study [27], which changed the pooled difference from [MD = 1.84, 95%CI: (− 1.49, 5.16), *p* = 0.28] to [MD = 2.64, 95%CI: (1.69, 3.59), *p* < 0.00001]. This result indicated that the pooled test performance of this analysis could be influenced by a single study. Therefore, the conclusion should be carefully drawn and further tested.

## Discussion

Serum OC has been widely applied to indicate bone turnover status in PMO management with several advantages such as bone specificity, non-invasiveness and dynamic-response. However, updated knowledge of OC challenges its traditional role as a bone turnover indicator in vivo, and studies have reported conflicting results of the change of sOC level in PMO cases.

PMO presents a high bone turnover rate with both increased bone resorption and formation [10–12]. Accordingly, most of the included studies (six out of ten) in our meta-analysis reported a significantly increased sOC level in PMO patients. Yet, there were also three studies reporting the same sOC level between cases and controls and even one study reporting a decreased sOC level in PMO cases.

The subsequent meta-analysis of all the included studies revealed only a trend of increase in sOC level in PMO cases compared to controls [95%CI: (− 1.49, 5.16), *p* = 0.28], which is not statistically significant. Subgroup analysis according to different regions and sOC fragments also revealed the same sOC level between cases and controls except in the European subgroup, where a significantly increased sOC level was observed in PMO patients.

By sensitivity analysis, we found that the pooled result was significantly influenced by Al-Daghri’s study [27], in which a significant decrease of sOC level was reported in PMO patients. We analyzed the Asian subgroup and found that after removal of Al Daghri’s study, the increase of pooled sOC level in Asian PMO cases compared to Asian controls became significant (from 0.98 to 0.03), just consistent with the result found in European subgourp. Therefore, it is possible that the different results reported in the subgroup analysis was not due to regional differences, but rather resulted from the effect of Al Daghri’s study on the Asian subgroup. However, every included study counts and we cannot ignore any study meeting the inclusion criteria yet with unexpected result. According to previous studies, osteocalcin, once undercarboxylated (unOC), had been found to act as a hormone [28] and had a board spectrum of interactions with glucose metabolism [29], fertility [30] and even aging [31]. In Al-Daghri’s study, the exact reason for the decreased sOC level in PMO cases remained unknown, but interactions of OC with glucose metabolism might have an effect on the result since fasting glucose levels in cases were slightly higher than controls and reached over 7.0 mmol/L (PMO vs control: 7.2 ± 3.6 vs 6.5 ± 3.2 mmol/L), indicating possible energy metabolism disorder in PMO group.

The major result of our current meta-analysis indicated no significant difference in the pooled sOC level between PMO cases and controls, which was consistent with the previous relevant meta-analysis reporting the same sOC level in osteoporotic and healthy populations in 2012 [13]. However, they included both primary (both type I and type II) and secondary osteoporosis, which might have introduced confounding factors into the analysis. In contrast, we compared only PMO cases with age and sex matched controls, in which the bone metabolic pattern was homogeneous and well clarified. Moreover, subgroup analyses according to the potential confounding factors (region of the study population and sOC fragment) were further performed. Therefore, our analysis was more specific and reliable to PMO. Besides, we have also compared the sOC levels in male population with and without OP in a previous meta-analysis and found no significant difference either [32].

However, some limitations should be mentioned in this meta-analysis. First, all the included studies were case-control studies due to the design of our research, and the level of evidence was lower than randomized controlled trials (RCTs). Second, significant heterogeneity existed between studies, which could still not be eliminated by subgroup analysis. The heterogeneity between studies might probably come from: 1. variations in the target sOC fragments evaluated by different assay kits; 2. variations in the experimental conditions [33, 34]. The intact OC molecule is rapidly degraded in serum, generating variable segments, thus limiting its utility in lab tests [35, 36]. Therefore, new techniques for sOC evaluation as well as standardization of methodologies and experimental conditions are urged for better application of sOC. Third, due to limited information from the included studies, physical activity levels could not be considered for variable control in our analysis, which could also have an effect on bone remodeling. However, there might not be a significant difference in physical activity levels in the senile postmenopausal populations between PMO cases and controls as long as the age and postmenopausal status were matched. Last but not least, the carboxylation status of sOC was not mentioned in any of the included studies. As a matter of fact, both OC and unOC in the circulation have been reported to correlate with each other [37, 38] and with bone quality [39, 40] but most of the conventional sOC assay kits are designed for total sOC while unOC can only be evaluated with special techniques [41, 42]. The result of this meta-analysis might not be significantly influenced by the carboxylation status of sOC as the same OC assay kit was adopted in PMO and control groups within each individual study.

## Conclusions

Based on the current available evidence, there is no significant difference of the pooled sOC level in PMO cases compared to postmenopausal controls. Since OC molecules are quite heterogeneous (different carboxylation status and different fragments) in the circulation and can be influenced by multiple metabolic events, sOC is not a good indicator for the high bone turnover status in PMO unless novel techniques for standardized circulatory sOC evaluation are applied in the future. Despite our rigorous methodology, the level of available evidence can still be improved with further high quality studies.

## Data Availability

The datasets used and/or analyzed during the current study are available from the corresponding author on reasonable request.

## Abbreviations

CI: Confidence interval
MD: Mean difference
N-MID: N-terminal mid-fragment of OC molecule
NOS: Newcastle-Ottawa Scale
OC: Osteocalcin
PMO: Postmenopausal osteoporosis
PRISMA: Preferred Reporting Item for Systemic Review and Meta-analysis
RCTs: Randomized controlled trials
sOC: serum osteocalcin
